# 

**Published:** 1859-10

**Authors:** 


☞ We would respectfully notify our Subscribers that we have REMOVED the Publication Office of this journal from the Everett House, to 884 BROADWAY. Our friends will be kind enough to address for the future to that location.

H. B. PRICE,
*Publisher and General Agent for Country Orders,*
884 BROADWAY, N. Y.

---

### REVIEWS, NOTICES, &c.

*Republication of the London, Edinburgh, North British and Westminster Quarterly Reviews*, $3.00 a year each. *Blackwood's Magazine*, monthly, $3.00 a year. The four Reviews and Blackwood, $10.00 a year; only by Leonard Scott & Co., 79 Fulton Street, N. Y.

*Dental Cosmos*, monthly, $2.50 a year; published by Jones and White, Philadelphia.

*The Happy Home and Parlor Magazine;* Boston, G. C. Stone & Co., Boston, Mass, $2.00 a year.

*The Home Monthly*, a household magazine, $1.50 a year; Buffalo, N. Y. Edited by Mrs. H. E. G. Arey, and Mrs. C. H. Gildersleeve.

*Godey's Lady's Book*, $3.00 a year; Philadelphia. Fifty-ninth volume. Richly and profusely illustrated.

*Presbytery Reporter*, monthly; Chicago, Illinois; $1.00 a year. Rev. A. T. Norton, editor.

*Presbyterian Expositor*, $1.50 a year; Chicago, Ill. Edited by Rev. N. L. Rice, D. D., Professor in the North Western Theological Seminary, under the care of the General Assembly of the Presbyterian Church in the United States of America, Old School.

*Michigan Journal of Education*, $1.00 a year; published monthly, at Ann Arbor, Michigan.

*Eclectic Medical Journal*, Philadelphia, $2.00 a year. Edited by William Paine, M. D.

*Boston Medical and Surgical Journal*, $3.00 a year, published monthly, and edited by Wm. W. Morland, M. D., and Francis Minot, M. D.

*Evangelical Repository*, Philadelphia, $1.50 a year. Edited by Thomas H. Beveridge, Pastor of the Sixth United Presbyterian Congregation.

*Medical and Surgical Reporter;* a weekly journal, $3.00 a year, Philadelphia. Edited by S. W. Butler, M. D., and R. J. Levis, M.D.

*Millenial Harbinger*, published monthly, at $1.00 year, by Alexander Campbell of Bethany, Virginia.

*Presbyterian Magazine;* Philadelphia, $1.00 a year, vol. 9. Edited by Rev. C. Van Rensellaer, D. D.

*Pacific Expositor*, published monthly, at $3.00 a year. Edited by Rev. Wm. A. Scott, D. D., Pastor of the Calvary Presbyterian Church, in San Francisco, California.

*Challen's Monthly*, $1.00 a year, Philadelphia; by James Challen & Son.

*Ladies' Magazine*, Philadelphia, $3.00 a year, published monthly, with fine illustrations.

